# Catheter-Based Ventricular Assist Device–Induced Severe Hyperthermia

**DOI:** 10.1016/j.jaccas.2026.110201

**Published:** 2026-09-02

**Authors:** Sarah Noorani, Tyson Burnham, Eric Zuniga, Antonio H. Frangieh, Andy Y. Lee

**Affiliations:** a Department of Medicine, University of California-Irvine Medical Center, Orange, California, USA; b Mary & Steve Wen Cardiovascular Division, University of California-Irvine Health, Irvine, California, USA; c Jeffrey M. Carlton Heart & Vascular Institute, Hoag Memorial Hospital Presbyterian, Newport Beach, California, USA

**Keywords:** acute coronary syndrome, acute heart failure, cardiac assist devices, cardiomyopathy, chest pain, drug abuse, hemodynamics, inotropes, left ventricle, left-sided catheterization

## Abstract

**Background:**

Hemolysis is a potential complication of catheter-based ventricular assist devices (CVADs), but severe hyperthermia is a rare occurrence that significantly affects further management and outcomes.

**Case Summary:**

A young man with toxic methamphetamine-associated cardiomyopathy presented with cardiogenic shock, prompting placement of a CVAD. He soon developed severe hyperthermia unresponsive to cooling measures. The hyperthermia ultimately subsided with removal of the device.

**Discussion:**

Hemolysis and renal failure are known complications of CVAD placement. The hyperthermia in this patient was likely related to erythrocytosis, highly viscous blood, and resulting hemolysis. The patient returned to normothermia only after removal of the device, supporting the idea that the CVAD was a cause of hyperthermic episode.

**Take-Home Messages:**

The use of CVADs in cardiogenic shock offers favorable outcomes but has potential complications. This unique case of severe hyperthermia resulting from massive hemolysis that required prompt removal of the device supports a rare, high-morbidity potential complication.

## Visual Summary

**Figure:**
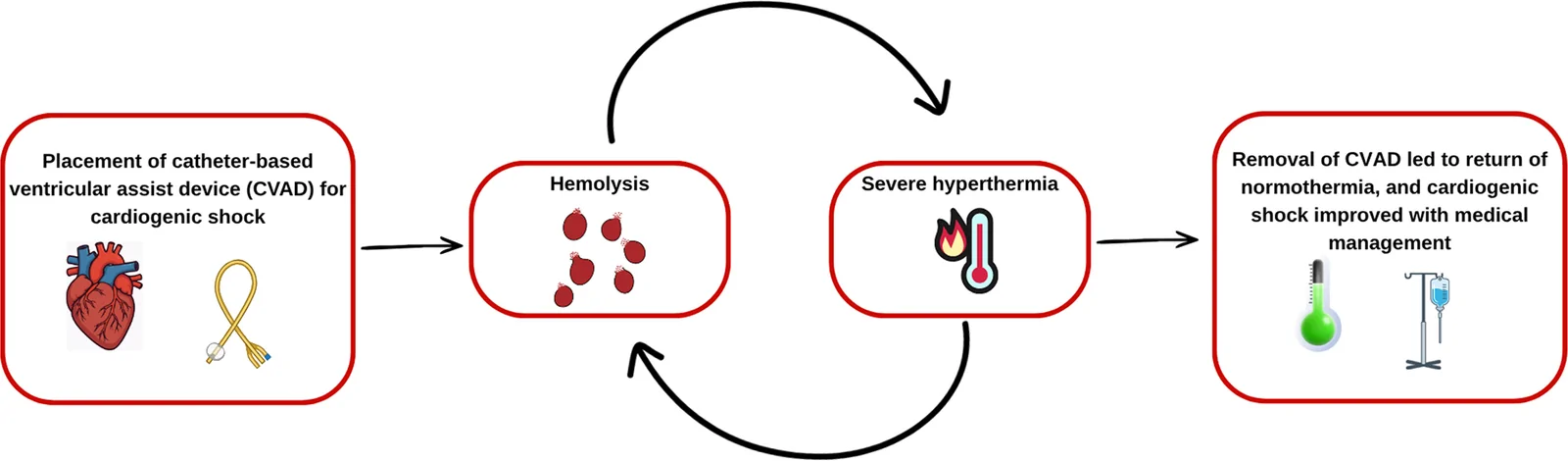

## History of Presentation

A 42-year-old man presented with acute onset of chest pain. The patient's vital signs were notable for tachycardia to 120 beats/min, blood pressure of 148/89 mm Hg, and normothermia with temperature of 37.1 °C. He was hypoxic to 87% on room air, so he was placed on 4 L nasal cannula with improvement in saturation to 100%.

## Past Medical History

The patient's medical history was notable for heart failure with reduced ejection fraction secondary to methamphetamine-induced cardiomyopathy.

## Differential Diagnosis

The differential included ST-segment elevation myocardial infarction, decompensated heart failure, acute valvular disease, or toxin-induced cardiomyopathy.

## Investigations

The workup was notable for troponin of 88 ng/L, white blood cell count 9.0 thousand/μL, hemoglobin 19.8 g/dL, brain natriuretic peptide 228 pg/mL, and electrocardiogram showing anterior ST-segment elevations (Figure 1).

**Figure 1:**
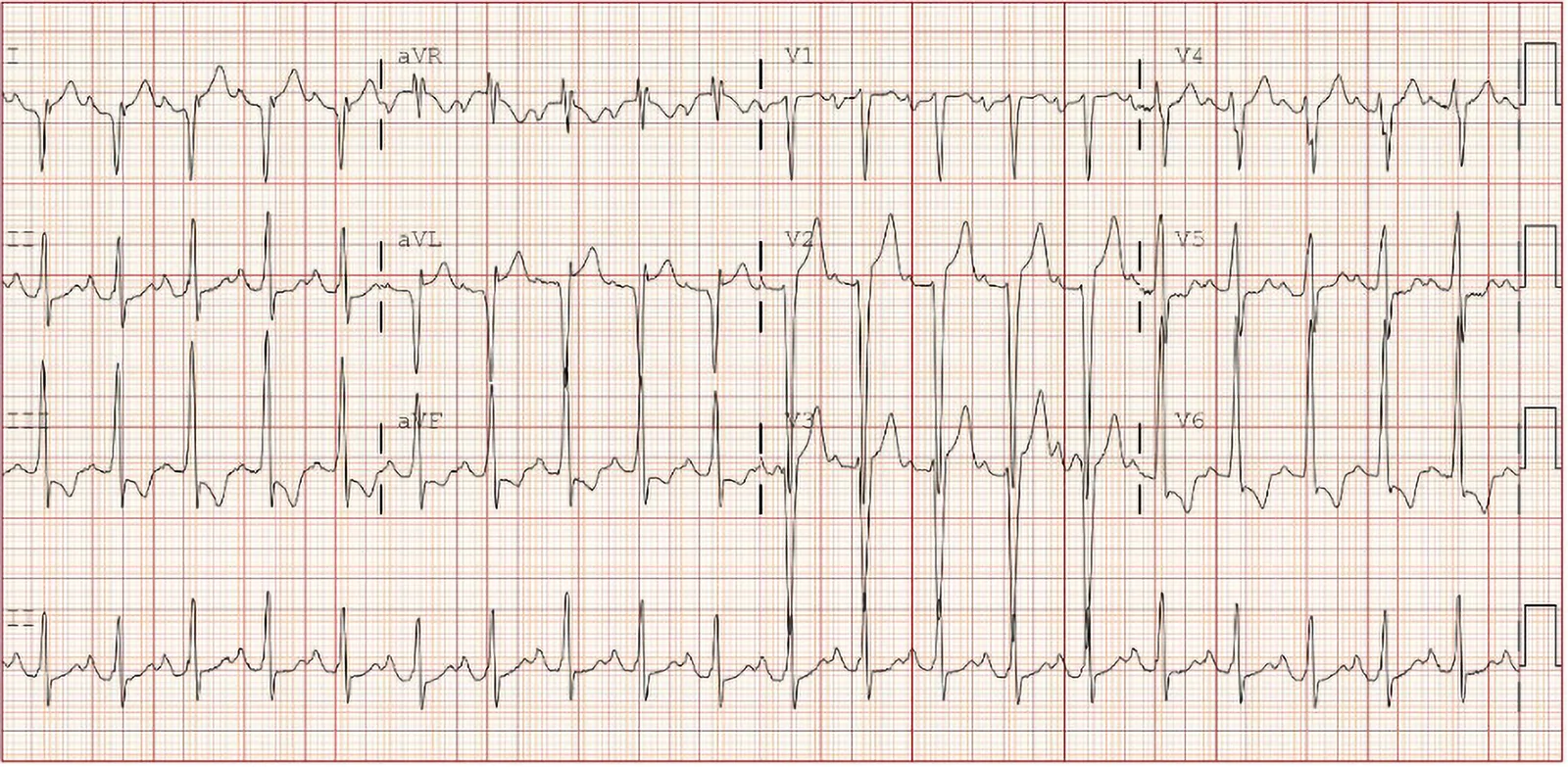
Electrocardiogram on Presentation Concerning for STEMI Electrocardiogram showing ST-segment elevations in the anterior leads as well as ST-segment depression and T-wave inversions in the inferior leads. STEMI = ST-segment elevation myocardial infarction.

The catheterization laboratory was activated for an ST-segment elevation myocardial infarction, and the patient underwent emergent left heart catheterization with fentanyl and midazolam used for analgesia and sedation. He was found to have mild nonobstructive coronary artery disease, with the most severe lesion being 30% stenosis of the proximal first diagonal branch of the left anterior descending artery. His left ventricular end-diastolic pressure was severely elevated to 42 mm Hg, which prompted placement of a Swan-Ganz catheter and a CVAD (Impella CP, Abiomed) (Figure 2). The measurements taken after CVAD placement included a mean right atrial pressure of 22 mm Hg, right ventricular pressure of 57/19 mm Hg, pulmonary artery pressure of 57/41 mm Hg with a mean of 47 mm Hg, mean pulmonary capillary wedge pressure of 26 mm Hg, pulmonary artery saturation of 58%, cardiac output by Fick of 3.09 L/min, and cardiac index by Fick of 1.39 L/min/m^2^.

**Figure 2:**
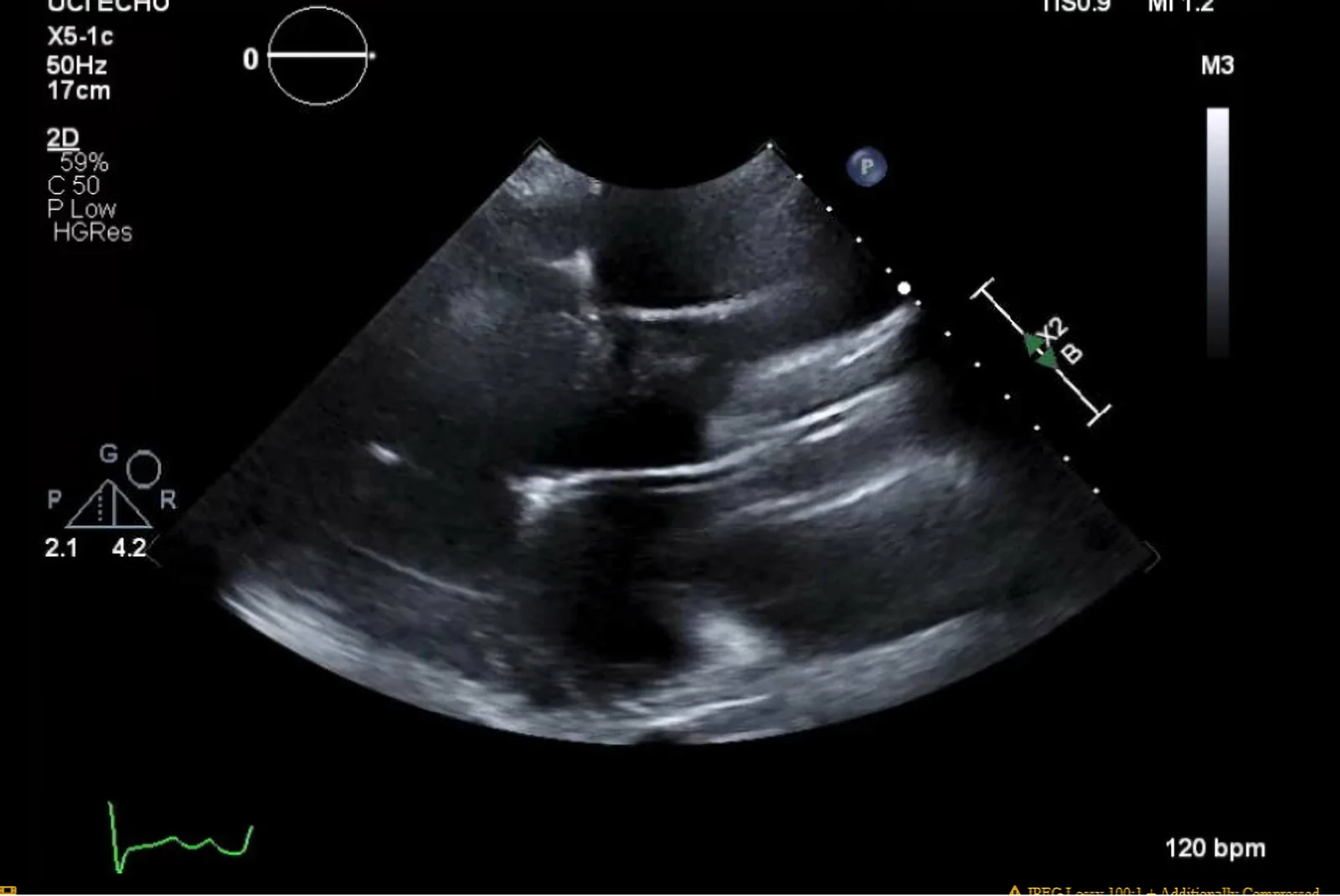
Echocardiogram Taken After CVAD Placement Echocardiogram demonstrating stable position of the CVAD across the aortic valve inlet. CVAD = catheter-based ventricular assist device.

## Management

Following CVAD placement, the patient demonstrated rapid improvement in cardiac index and filling pressures. However, within the next hour he developed gross hematuria and severe hyperthermia, with temperatures reaching a maximum of 40.7 °C. Repeat laboratory tests were drawn and revealed a decreasing hemoglobin and elevated indirect bilirubin concerning for hemolysis. Additional hemolysis work-up, including lactate dehydrogenase and haptoglobin, was unable to be performed because of exceeded hemolysis indices. Blood work was also notable for an acute kidney injury, as evidenced by a creatinine level of 4.0 increased from 1.6 on admission, creatine kinase 898 U/L, potassium 4.3 mmol/L, and pH 7.35. Infectious work-up was unremarkable thus far, but the patient was started empirically on piperacillin-tazobactam. Blood cultures were drawn and ultimately had no microbial growth. Cooling measures were rapidly initiated, to which the patient did not substantially improve. As the patient remained profoundly hyperthermic, he was intubated for insertion of an esophageal cooling device, using etomidate and rocuronium for induction and noting an end-tidal co_2_ of 40 mm Hg. Although these measures initially reduced the temperatures, the effect was transient, and his severe hyperthermia returned. As the favored potential etiology for the patient's acute hyperthermia was the CVAD, the shock team elected to remove the CVAD later the same day. After removal of the device, the patient's hyperthermia rapidly resolved (Figure 3). To further manage the patient's cardiogenic shock, he was started on dobutamine and norepinephrine.

**Figure 3:**
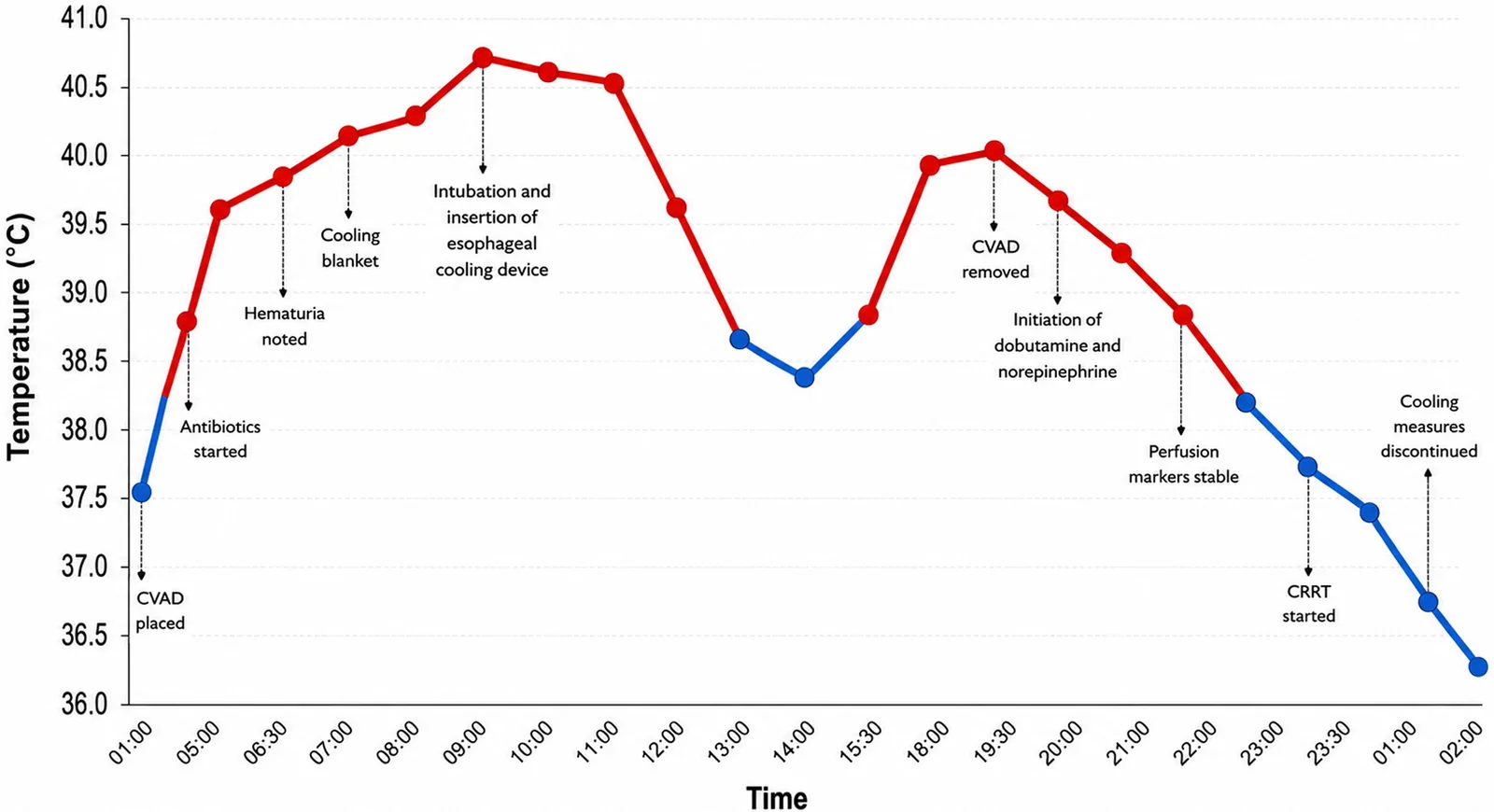
CVAD Placement Associated With Hyperthermia Graph of temperature measured over time demonstrating temperatures up to 40.7 °C after CVAD placement. Implementation of cooling measures, including cooling blanket and esophageal cooling device after intubation, reduced temperatures but failed to achieve normothermia. Removal of the CVAD led to rapid reduction of temperatures back to normal over the next few hours. CRRT = continuous renal replacement therapy; CVAD = catheter-based ventricular assist device.

## Outcome and Follow-Up

With supportive care and volume removal via continuous renal replacement therapy, the patient's cardiogenic shock resolved. Once clinically stabilized, he was discharged with arrangements for outpatient hemodialysis and follow-up with nephrology and cardiology. He received dialysis 3 times weekly for 2 months, but this was ultimately discontinued given eventual renal recovery. The patient did not follow up with the cardiology team in clinic and continued to use methamphetamine with multiple hospital readmissions for overdose and heart failure exacerbations.

## Discussion

Early recognition of cardiogenic shock and initiation of aggressive therapies has gradually reduced the mortality rate over time.^1^ Despite these improvements, the in-hospital mortality of patients presenting with cardiogenic shock remains high, with estimates between 40% and 60%.^1^^,^^2^ CVADs are increasingly used to manage cardiogenic shock or high-risk percutaneous coronary intervention.^3^ These devices significantly increase cardiac output, decrease filling pressures, and improve coronary perfusion, thus reducing the need for pharmacologic support in cases of cardiogenic shock.^2^ The use of CVADs has been increasing, whereas the use of intra-aortic balloon pumps has been declining given superior hemodynamic parameters with CVADs compared to intra-aortic balloon pumps, including cardiac output and mean arterial pressure.^3^^,^^4^

In the present case, we used the Impella CP, which is a microaxial pump positioned across the aortic valve into the left ventricle.^4^ It provides continuous anterograde flow from the left ventricle into the aorta, thus reducing the workload of the left ventricle, increasing cardiac output, and improving both systemic and coronary perfusion.^4^ Common complications include bleeding, left ventricular perforation, stroke, and sepsis.^5^ Hemolysis, hematuria, and renal failure are known but less frequent complications of CVAD placement that are more commonly seen in the setting of cardiogenic shock rather than percutaneous coronary intervention.^6^ The resulting hemolysis is most likely due to the small impeller rotating at a high speed inside the thin catheter, which places high shear stress on blood cells, causing hemolysis and potentially acute renal failure due to pigment nephropathy.^7^ Prior studies have also found the occurrence of postimplantation hyperthermia in response to CVAD placement. The majority of these cases have failed to identify an infectious source, suggesting that mild hyperthermia may be an inconsequential result of CVAD placement that does not require intervention.^8^

Device-related factors that increase the risk for hemolysis include higher pump support levels, notably P7 and P8, pump thrombosis, and improper positioning of the device.^7^ Moreover, the type of CVAD also plays a role in the development of hemolysis. The Impella CP has been associated with a higher incidence of hemolysis compared to the Impella 5.0/5.5.^9^ One study also found that replacing the Impella CP with the Impella 5.5 after the onset of hemolysis effectively reduced the hemolysis, prevented further renal damage, and improved clinical outcomes.^9^ Patient-dependent factors that increase hemolysis risk include smaller left ventricular end diastolic volume or low preload due to hypovolemia, vasoplegia, or right ventricular failure.^7^ Other factors such as blood transfusions, sickle cell anemia, autoimmune hemolytic anemia, or surgery may also contribute to hemolysis.^7^

In the present case, the patient rapidly developed hemolysis within 4 hours after CVAD placement, which is a relatively quick time of onset compared to prior studies, which found the median time of onset to be 16.5 hours.^9^ The only identifiable risk factor for hemolysis was erythrocytosis; all other device-associated and patient-associated risk factors had been addressed to minimize the risk. The patient was only on performance level P2, the device was in the proper position as determined by imaging both postprocedure and after the onset of hemolysis, and he was hypervolemic as evidenced by an elevated left ventricular end-diastolic pressure. There were no suction alarms, and while pump thrombosis was considered, this was deemed to be less likely given therapeutic anticoagulation, normal purge pressures, and normal flow.

The rapid onset of hemolysis was accompanied by significant hyperthermia temporally associated with insertion of the CVAD. Other potential causes of severe hyperthermia were also considered but were less likely, including infection, malignant hyperthermia, rhabdomyolysis, and methamphetamine-induced hyperthermia. Infectious work-up was negative, there was no use of volatile anesthetics or succinylcholine, and the mild elevation in creatine kinase was not consistent with rhabdomyolysis. Given this patient's history of methamphetamine use, drug-induced hyperthermia is another possibility but is less likely given the patient's hypotension rather than the classic hypertension seen in such cases; failure to respond to cooling measures and midazolam, which is the mainstay of treatment for that condition; and rapid normalization of temperatures with removal of the CVAD. While the exact mechanism remains unknown, CVADs have been found to be associated with elevated levels of proinflammatory cytokines, including interleukin-6 and tumor necrosis factor–alpha, suggesting that these devices induce an inflammatory state.^10^ In this patient with erythrocytosis, highly viscous blood, and resulting hemolysis, it is likely that the severe hyperthermia was a consequence of large-volume CVAD-induced hemolysis. The hyperthermia rapidly subsided after the removal of the CVAD, which is a supportive finding of this unusual symptom.

## Conclusions

CVADs are valuable tools in treating patients with cardiogenic shock but are associated with various complications, including bleeding, left ventricular perforation, sepsis, and hemolysis. This case highlights a unique case of severe hyperthermia associated with CVAD placement and the resulting hemolysis. There was no infectious etiology or risk factors for malignant hyperthermia identified, and the hyperthermia immediately resolved after removal of the CVAD. Although the exact mechanism remains unknown, recognizing CVAD-associated hyperthermia is crucial, as this unusual symptom necessitates removal of the CVAD and limits management of cardiogenic shock to inotropic support.

**Visual Summary:**
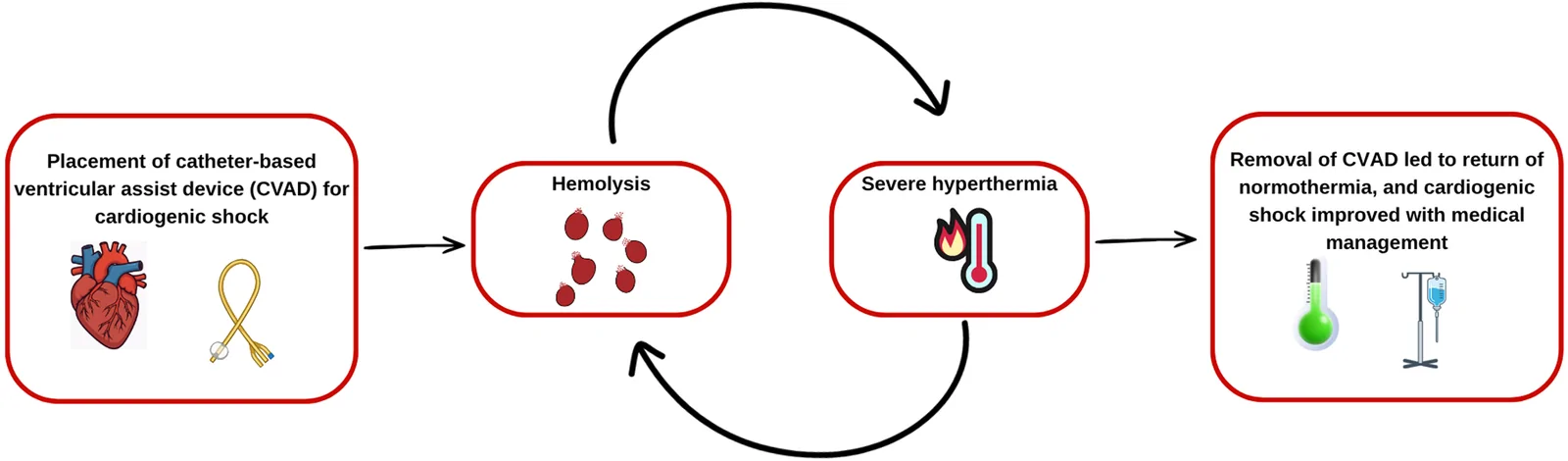
CVAD-Induced Hyperthermia in Cardiogenic Shock Placement of a CVAD for cardiogenic shock led to improved hemodynamics but was associated with severe hyperthermia, with temperatures returning to normal after prompt removal of the device. CVAD = catheter-based ventricular assist device.

## Funding Support and Author Disclosures

The authors have reported that they have no relationships relevant to the contents of this paper to disclose.

**Take-Home Messages**

• The use of catheter-based ventricular assist devices (CVADs) is an effective modality to improve cardiac output in cases of cardiogenic shock, and while there are several well-known complications, severe hyperthermia is a rare finding that significantly impacts management.
• Early recognition of CVAD-associated severe hyperthermia is crucial as it requires prompt removal of the device and appropriate medical management.

**Equipment List:** 

Type of Equipment	Specifics
Access	Introducer Sheath 6 F × 2.5 cm × 10 cm Pinnacle (Terumo Medical)
	Tip Echo 7 cm Needle Nitinol GW Stiffen Fluent
	Kit Micro-Introducer 5 F × 10 cm Straight (Galt Medical)
	Fixed Core Wire Guide Safe (Cook Medical)
	Catheter Angiographic 5 F × 100 cm (Cardinal Health)
	Swan-Ganz VIP Oximetry Thermomodulation Catheter 7.5 F × 110 cm (Edwards Lifesciences)
Ventricular assist device	Ventricular Assist Device Impella CP Pump (Abiomed)
Closure	Vascular Closure Perclose (Abbott)
